# Fecal Microbiota Transplantation: Expanding Horizons for *Clostridium difficile* Infections and Beyond

**DOI:** 10.3390/antibiotics4030254

**Published:** 2015-07-06

**Authors:** Thomas J. Borody, Debra Peattie, Scott W. Mitchell

**Affiliations:** 1 Centre for Digestive Diseases, 1/229 Great North Rd, Five Dock, NSW 2046 Australia; E-Mail: scott.mitchell@cdd.com.au; 2 Pleiades Advisors, 13 Oak Meadow Road, Lincoln, MA 01773, USA; E-Mail: debra.peattie@pleiadesadvisors.com

**Keywords:** Fecal microbiota transplantation, *Clostridium difficile*, inflammatory bowel disease, IBD, colitis, Crohn’s, irritable bowel

## Abstract

Fecal Microbiota Transplantation (FMT) methodology has been progressively refined over the past several years. The procedure has an extensive track record of success curing *Clostridium difficile* infection (CDI) with remarkably few adverse effects. It achieves similar levels of success whether the CDI occurs in the young or elderly, previously normal or profoundly ill patients, or those with CDI in Inflammatory Bowel Disease (IBD). While using FMT to treat CDI, however, we learned that using the procedure in other gastrointestinal (GI) diseases, such as IBD without CDI, generally fails to effect cure. To improve results in treating other non-CDI diseases, innovatively designed Randomized Controlled Trials (RCTs) will be required to address questions about mechanisms operating within particular diseases. Availability of orally deliverable FMT products, such as capsules containing lyophilised fecal microbiota, will simplify CDI treatment and open the door to convenient, prolonged FMT delivery to the GI tract and will likely deliver improved results in both CDI and non-CDI diseases.

## 1. Introduction

This review avoids detailed summary about Fecal Microbiota Transplantation (FMT), a procedure that introduces normal donor colonic microbiota in the form of blended stool into a recipient’s bowel to treat recurrent *Clostridium difficile* infection (CDI). The efficacy of FMT to treat recurrent CDI was initially proven in a randomized controlled trial by Van Nood *et al*. [1], where the study was halted due to the overwhelming success of donor FMT compared with vancomycin. Here, we reference “headline” topics in FMT in the Introduction, focusing primarily on CDI situations facing clinicians, questions arising among research physicians and emerging changes in compositions and delivery formats of transplantation material for FMT.

Since its first detailed description in 1958 by Ben Eiseman [2] using FMT in staphylococcal pseudomembranous enterocolitis, retrospectively thought to be CDI, FMT has matured through various stages. This process has involved the following:

- *Early Case Reports*: The “discovery phase” stemmed from a number of literature reports, often expressing considerable surprise that FMT worked so well to dramatically cure terminally ill patients. The severe condition was thought to be a staphylococcal pseudomembranous enterocolitis [2,3,4,5], but after 1981 [6,7], when CDI toxin became detectable [3,4,5,6,7], clinicians questioned and minimized the role of *Staphylococcus aureus* in pseudomembranous intestinal lesions because they realized that most pseudomembranous enterocolitis diagnoses were positive for CDI toxin [8].
- *Donor Selection*: Half a century later, with FMT becoming used more frequently, a group experienced in utilising FMT published the first standardized donor selection guidelines [9]. More stringent selection criteria are already appearing, and there is every reason to believe that more refined guidelines will emerge as we incorporate additional exclusion criteria, such as family history of obesity, metabolic syndrome and diabetes [10], into FMT practice.
- *Methodology Refinements*: As FMT case reports publish from various parts of the world, summaries and comparisons of numerous methodological analyses are helping refine FMT practice [11,12,13,14]. Topics reviewed to date include history of FMT, the changing face of CDI and stool solvent, volume and delivery routes (via the upper GI tract *versus* the lower GI tract). For example, and perhaps significant for future FMT administration, a recent study reported 27 patients who underwent fixed volume FMT delivered via *upper enteroscopy and colonoscopy at the same session* to cover both the small and large intestine. In the largest study of its kind, all 27 patients cleared *C. difficile* toxin from their systems, resulting in 100% clinical efficacy with only minimal, transient adverse effects [15]. These cure rates contrast with that of 73%–92% for delivery via nasogastric/duodenal tubes and via upper endoscopy. This “dual coverage” method points to the importance of developing encapsulated microbiota to treat CDI, so that normal microbiota can be administered via oral delivery, thereby exposing the entire GI tract to healthy, diverse flora and potentially improving efficacy of CDI eradication.
- *Long-Term Follow-up*: Several articles have addressed the need for long-term follow up of patients receiving FMT since it is crucial to know about any untoward effects that may only develop long after the procedure [14,16]. At this time, short-term adverse effects have been remarkably infrequent following FMT [11]. In a long-term follow-up study of 77 patients after FMT, some new diseases developed in 4 subjects, although these had not been present in the donors and a clear relationship between the new disease and FMT was not evident [17]. Reflecting on the origin of the microbiota used in FMT, one could conclude that this “biologic” therapy derived from a healthy donor, say 35 years of age, has already undergone multiple decades of *in vivo* “testing” for adverse events within that healthy donor [18]. Long-term safety evidence to date [17] implies that microbiota from healthy, well-screened donors is currently the safest FMT product we have, but longitudinal studies to monitor and correlate gut microbiota with the health of donors and recipients have yet to be done. Indeed, in our own practice with 26 years follow-up of several thousand FMT recipients, there has been no donor-to recipient illness transfer. Ironically, the perceived safety of cultured consortia of defined microbiota, with their inherent potential for gene transfer and exchange, cannot rival the deep level of safety that comes from a healthy, well-screened human donor. Such data come from treating with non-toxigenic *C. difficile* to outcompete toxigenic strains to prevent colonization [19]. The *C. difficile* toxin-encoding PaLoc region from a toxigenic strain was recently shown to mobilize to non-toxigenic isolates, indicating that non-toxigenic strains can become toxigenic through horizontal gene transfer events [20]. Using spores of non-toxigenic strains, in this case *Clostridia* spores*,* in therapeutics may be risky, as evidenced by the fact that several placebo patients were found to be infected with non-toxigenic strains during clinical trials, apparently due to spore contamination of communal areas [21].
- *Regrowth of Depleted Microbiota*: Pre- and post-FMT microbiota composition of the recipient’s GI tract has also been of particular interest. While it is possible to achieve durable implantation of donor bacteria [22], it is also becoming clear that eradicating *C. difficile* by antagonistic, but non-pathogenic, *Clostridium* spores can lead to a recovered, functional microbiota population due to regrowth of occult “missing” components such as *Firmicutes* and *Bacteroidetes* [23].
- *FDA Oversight*: In March 2014, the United States Food and Drug Administration (FDA) announced its intention to exercise enforcement discretion regarding Investigational New Drug (IND) applications for use of FMT for recurrent CDI. An IND application is not required to treat recurrent CDI cases, but it is still necessary for non-CDI indications and for research situations. Understanding the FDA position is crucial both to clinicians and FMT research teams [24].

## 2. Expanding Use of FMT for CDI in Specialised Clinical Situations

As more hospitals and clinics have begun using FMT to treat CDI, questions of safety and efficacy have arisen about special patient groups. We address these below using individual publications that combine cases from several practitioners.

- *Elderly Patients*: Elderly patients are the most susceptible to relapse after initial treatment of CDI with standard of care antibiotic therapy [25,26] and are the majority of patients currently treated for relapsing CDI. Consequently, it is important to know that FMT is safe and effective in this patient population. Using data collected from multiple centres, Agrawal *et al*. [27] reported on 146 patients, finding 83.5% and 95.2% primary and secondary cure rates in relapsing CDI. These rates associated with a short-term adverse effect rate of 3.4% either due to the CDI, the FMT or both.
- *Patients with Severe CDI*: The only published study with indices about this indication is a collection of 13 cases from a multicentre study of severe and/or complicated CDI in patients who had failed several courses of antibiotics and who were subsequently treated with FMT. Of these, 84% had severe CDI and 92% had complicated CDI, and their mean post-treatment follow-up was 15 months. Primary and secondary cure rates were 84% and 92%, respectively, with minimal adverse effects of abdominal bloating and cramping early post treatment [28]. Such data indicate that age and severity should not be a barrier when considering FMT as a treatment option in the elderly, even those with severe and complicated CDI.
- *The Immunosuppressed*: This unique patient group frequently contracts CDI, and concerns have arisen regarding the safety of FMT for immunosuppressed patients with IBD and non-IBD associated-CDI given the possibility that septicaemia could result from the FMT procedure. In a retrospective multicentre study, Kelly *et al*. [29] reported on 80 CDI patients, each immunosuppressed due to HIV, solid organ transplant or cancer, and 36 of whom also had IBD. All patients received FMT, resulting in an 89% cure rate of CDI without any infection resulting from the FMT. They recorded several treatment-related adverse effects, including sedation-related aspiration and worsening of IBD. Brandt *et al*. [30] reported on a smaller cohort of 13 immunosuppressed IBD patients who had no CDI but were being treated for IBD with FMT. Apart from transient abdominal distension and bloating in 2 patients, there were no other adverse effects. Such data reinforce the conclusion that immunosuppressed patients have no increased risk of infection from the FMT treatment itself relative to non-immunosuppressed patients.
- *Patients after Sub-total/total Colectomy*: There are, as yet, few publications to indicate whether patients with partial or total colectomy suffering CDI are more difficult to cure with FMT than other types of CDI patients. At the Centre for Digestive Diseases (CDD), we have had a total of 3 patients with sub-total colectomy for whom FMT failed to cure their CDI, even with combined, repeated nasojejunal and colonic transcolonoscopic infusions; these patients have continued to have CDI for up to 7 years. There has been one publication [31], however, in which FMT via nasoduodenal administration cured a patient after total proctocolectomy of CDI in the remaining small bowel.
- *Patients with IBD and CDI*: A proportion of patients with ulcerative colitis (UC) and Crohn’s disease (CD) are co-infected with *Clostridium difficile*. We have reported treating such patients with FMT, noting efficient eradication of the CDI, but there are several outcomes for the IBD [32,33]. IBD symptoms may improve initially in a sub-group for the first few weeks/month, followed by symptom recurrence. In a larger proportion of patients, symptoms remain unchanged, and, in a small percentage, the IBD symptoms worsen, perhaps due to the withdrawal of antibiotics in patients who receive FMT (to avoid interfere with the implanted bacteria). Others have reported similar observations [34].

## 3. Expanding the Use of FMT to Non-CDI Colitis

**Figure 1:**
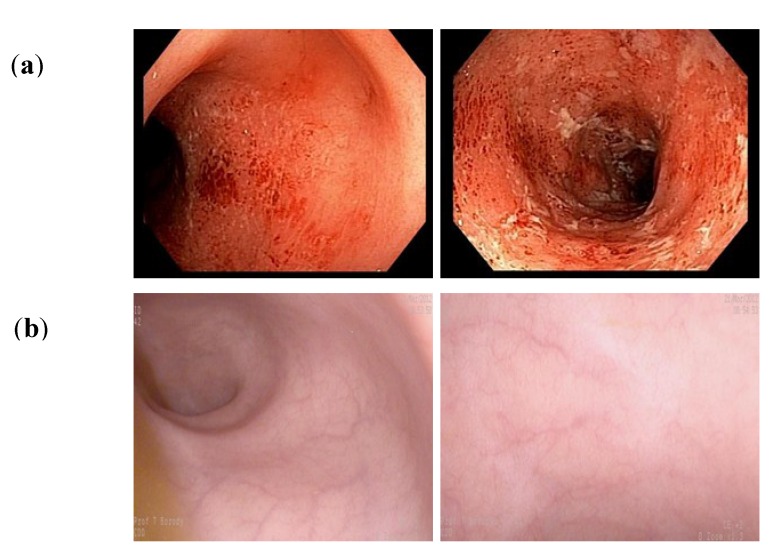
Sporadic “remission” of ulcerative colitis after 14 days of Fecal Microbiota Transplantation (FMT) showing mucosa before and three years after treatment. Patient off medications. (**a**) BEFORE FMT: Marked inflammation in rectum and sigmoid colon; (**b**) AFTER FMT: Absence of inflammation in rectum and sigmoid colon.

*Clostridium difficile* can be eradicated in patients with IBD even though the IBD is rarely cured. Whilst occasionally curable, IBD can respond to FMT, especially if the procedure is administered repeatedly, and can result in a “remission”. In 1988, we administered FMT to a patient at CDD for colitis in the absence of CDI — the first of such patients to receive FMT at our facility. Her indeterminate colitis completely disappeared over several weeks and has not recurred over the past 26 years of follow-up [35,36]. We term such profound IBD remission as a “Sporadic Remission” after FMT. Figure 1 documents a more recent example of a CDD patient who had 14 days of FMT, after which her colitis reversed completely to normality for 3 years even though she did not have CDI. Based upon our extended experience over 24 years of using FMT in colitis patients [37], we believe that FMT researchers, as a group, can modify treatment paradigms to achieve better *cure* results and not just short term *remissions*. The first randomized clinical trials of FMT in Ulcerative Colitis (UC) have now been published [38,39]. Moayyedi *et al*. [38] reported significant induction of remission with FMT in UC where 75 patients received either a 50 mL FMT via enema (n = 38) or 50mL water enema (n = 37) once weekly for six weeks. By week 7, 9/38 patients on FMT (24%) were in remission due to “donor effect” while only 2/37 on placebo (5%) were in remission (*p* < 0.03). In the second trial by Rossen *et al*. [39], patients received FMT via nasoduodenal tube at 0 and 3 weeks from either a donor (active arm) or their own stool (control arm). On intention-to-treat analysis, 7/23 on active (30.4%) and 5 of 25 controls (20.0%) achieved the primary endpoint (*p* = 0.51). Moayyedi achieved remission in spite of low FMT volumes, but his group did use frequent infusions via enema route. Rossen may have failed because only two infusions were carried out, and even these were via nasoduodenal route unlike the previously published UC methods, reflecting a trial design influenced by their previous CDI results [39]. Both trials measured attainment of remission rather than cure*,* yet they were testing a CDI therapy that measures *cure* rather than remission*.*

There is a fundamental difference between treating relapsing CDI with FMT and treating IBD with FMT. Relapsing CDI clearly has a different pathogenic mechanism than IBD, and outcome after FMT differs significantly between these two conditions. Perhaps in part due to publications documenting dramatic examples of sporadic remission in IBD after FMT (often actual cure of IBD [40]), pressure has been building to develop protocols, find funding and conduct randomized controlled trials (RCTs) for FMT in IBD. As recently noted, “*the sparse results reported for cases of IBD have been variable with regard to the success rate for inducing remission, and well-designed randomized controlled trials are currently still lacking*” [41]. RCTs for CDI aim to obtain cure with a single or several FMT infusions compared to placebo. Regrettably, not even well-designed RCTs will provide answers concerning IBD ‘cure’ until we throw out the CDI rule book and pose the correct questions for IBD. RCTs for IBD can be designed to give *short-term remission* following repeated administrations compared to currently used drugs such as steroids, but this approach does not result in prolonged remission. Alternatively, and taking “the high road”, we can try to emulate CDI results and maximize IBD cure as in Figure 1. As of late April 2015, there were at least 23 active trials listed on www.clinicaltrials.gov comparing FMT with placebo in IBD (Table 1). We predict that many of these trials will fail to show a significant difference between FMT and placebo, as seen in Rossen 2015 [39], because the intervention protocol used is that for CDI.

**Table 1:** Current and upcoming clinical trials targeting IBD and related illnesses with FMT

Clinicaltrials.gov Identifier	Indication being Trialed	Phase of Trial
NCT01790061	UC	Phase 2/Phase 3
NCT01793831	CrD	Phase 2/Phase 3
NCT01847170	IBD	Phase 1
NCT01896635	UC	Phase 2
NCT01947101	UC	Phase 1
NCT02016469	IBD	NP
NCT02033408	IBD	Phase 4
NCT02049502	UC-associated Pouchitis	Phase 2
NCT02058524	UC	Phase 1
NCT02092402	IBS	NP
NCT02108821	IBD	Phase 1
NCT02154867	IBS	Phase 2
NCT02199561	CrD	Phase1/Phase 2
NCT02227342	UC	Phase 1/Phase 2
NCT02291523	UC	NP
NCT02299973	IBS	NP
NCT02328547	IBS	Phase 2
NCT02330211	Crohn’s Colitis	Phase1/Phase 2
NCT02330653	UC	Phase1/Phase 2
NCT02335281	IBD	Phase 2
NCT02391012	IBD	Phase 1
NCT02417974	CrD	Phase 2
NCT02390726	UC	Phase 0

Abbreviations List: CDI–*Clostridium difficile* Infection, CrD–Crohn’s Disease, IBD–Irritable Bowel Disease, IBS–Irritable Bowel Syndrome, NP–Not Provided on clinicaltrials.gov, UC–Ulcerative Colitis

Hence, the alternative is to design FMT trials for IBD that address questions about what methods will improve FMT therapy to achieve more “sporadic remissions” of IBD. A clear example of such evolving methodology occurred when Dr. Patrizia Kump and colleagues initially trialed single FMT for UC and found no clinical improvement [42]. Later they used multiple FMT infusions, which achieved significant improvement from baseline [43], and noted that serially repeated FMT produced better efficacy than a single FMT [43]. Such informed trial designs are needed to drive improved outcomes. Issues to address may include the following:

- Number and frequency of FMTs
- Use of frozen stool *vs*. fresh stool *vs*. a selected consortium of organisms
- Pre-treatment with antibiotics, including which antibiotics, how many antibiotics and how long to pre-treat with antibiotics
- Determining whether there are more/less efficacious donor microbiota and what compositional differences affect efficacy
- Determining whether to administer FMT during active mucosal inflammation or to heal the mucosa with anti-inflammatory medications, e.g., immunosuppressive therapy, and then administer FMT
- Determining whether to maintain mucosal healing therapy after FMT, e.g., with 6-mercaptopurine or azathioprine for prolonged periods of time, while waiting for the transplanted microbiota to effect the “healing process” and, perhaps, to improve implantation? In a study by Borody *et al*. [37], patient follow up at 33 months revealed that 57% of the patients achieved mucosal healing when some were maintained on 6-mercaptopurine or azathioprine after FMT. This suggests that long-term follow-up of these patients, some of whom could be kept on anti-inflammatory agents, could be yet another mechanism by which to increase the success rate of FMT for UC patients.

## 4. FMT Use in Non-CDI Single Infections

Based on the success of FMT in treating CDI, clinicians have begun using FMT to treat *single* bacterial infections distinct from CDI. Singh *et al*. [44] recently described using FMT to eradicate an Extended Spectrum beta-Lactamase (ESBL) producing *Escherichia coli* from the colon of a renal transplant patient, leading to cessation of the patient’s recurrent urinary infection with this pathogen. We agree with the authors’ hypothesis that donor feces infusion can be effective against pathogens other than *C. difficile,* such as the ESBL-producing *Enterobacteriacae* of the large bowel. Due to the success they observed in this case, the authors have initiated a clinical trial using FMT in patients infected with ESBL-producing *Enterobacteriacae*. 

A second infectious agent treated successfully by FMT was reported by Freedman in 2014 [45]. In this report presented at the 2014 Infectious Diseases Week meeting in Philadelphia, Freedman described multi-drug resistant carbapenemase-producing *Klesbiella pneumoniae* residing in the bowel that caused repeated blood, joint and femur osteomyelitis infections in a 13-year-old girl. After all antibiotic treatments failed, FMT cleared her gut of this *Klebsiella* and further systemic infections over the next 5 years. This experience indicates that FMT can cure GI pathogens other than *C. difficile* and that such pathogenic bacteria may be a source of systemic infection. 

## 5. Microbiome-Derived FMT Therapies Moving to Mainstream Medicine

We believe that a broad array of microbiome-derived, or microbiome-inspired, therapeutics will be developed over the next decade for numerous indications, including both gastrointestinal and systemic disorders and diseases. The ability to cure recurrent *Clostridium difficile* infection (R-CDI) safely and effectively with FMT in a clinical setting has spurred several companies to pursue FDA-approvable therapeutics to treat that indication. To date these therapeutic candidates, which all, to the best of our knowledge, derive from the stool of selected, extensively tested healthy human donors, fall into three categories: (1) homogenized and minimally refined whole stool, thereby comprising a “full-spectrum” of gastrointestinal microbiota, (2) highly concentrated and quantitated “full-spectrum” microbiota and (3) highly concentrated and quantitated “narrow-spectrum” spores and/or vegetative cells. Table 2 lists current product candidates, the targeted routes of administration, and companies developing the products. The Office of Vaccines Research and Review (OVRR) within the FDA’s Center for Biologics Evaluation and Research (CBER) will evaluate each candidate as a biologic.

**Table 2:** Current FMT product candidates for various conditions.

Product Candidate	Route of Administration	Company
MB-101	Oral delivery	Assembly Biosciences^1^
Full-spectrum Microbiota^TM^	Oral, colonoscopic delivery	CIPAC Limited
RBX2660	Enema delivery	Rebiotix Inc
Ecobiotic® SER-109	Oral delivery	Seres Health

^1^ Collaborating with OpenBiome, a nonprofit 501(c)(3) organization

In 2012, Hamilton *et al*. [46] reported on 43 consecutive patients with recurrent CDI who were treated with fresh or frozen filtered material prepared as fecal bacteria extract. Following a single infusion, 90% of patients administered frozen donor material were cured of CDI. A preliminary study performed by Youngster *et al*. [47] assessed the safety and efficacy of encapsulated frozen FMT for relapsing CDI. Resolution of diarrhea was observed in 70% of patients after a single capsule-based FMT, with non-responders retreated, to achieve an overall 90% rate of clinical resolution of diarrhea. No serious adverse events were reported, and only minor transient events were noted, including mild abdominal bloating and cramping in 30% of patients, which resolved within 72 hours. While the bulk of material used for FMT procedures to date has been crude or refined suspensions of fresh or frozen fecal microbiota, issues of convenience and stability clearly demand development of lyophilised preparations as approved therapeutic products. 

Lyophilised product candidates to treat R-CDI are being developed in both the full-spectrum and narrow-spectrum categories; such formulations could be resuspended for nasogastric or rectal (enema or colonoscopy) delivery or encapsulated for oral administration. Encapsulated, orally administered microbiota therapies — whether narrow- or full-spectrum — will facilitate treating recurrent *C. difficile* infection and will expand our ability to explore safety and efficacy in other, non-CDI indications. Given the documented efficacy of fecal microbiota in curing R-CDI and non-CDI single infections, the positive — albeit inconsistent — effects on UC and Crohn’s disease and the growing awareness about the systemic importance of healthy gut microbiota [48], future clinical trials will likely explore microbiome-based therapies for a variety of other, non-gastrointestinal conditions. 

Susceptibility to obesity, liver disease, cardiovascular disease and malignancy correlate with gut microbiota dysbiosis [17], and specific bacterial populations have been identified in a variety of other disorders. For example, Longstreth *et al*. [19] have hypothesized that a motor neuron toxin produced by a *Clostridial* species causes sporadic amyotrophic lateral sclerosis (ALS) in susceptible individuals, Scher *et al*. [20] have found that an expanded population of intestinal *Prevotella copri* correlates with enhanced susceptibility to arthritis, while Hsiao *et al*. [21] have determined that gut microbiota modulate behavioral and physiological abnormalities associated with neurodevelopmental disorders such as autism spectrum disorder (ASD).

## 6. Conclusions

The use of FMT in CDI has become rapidly accepted as a simple yet effective therapy that is becoming frequently utilized by the medical community. Success in treating CDI with healthy microbiota has created a natural extension into exploring FMT therapy for other gastrointestinal conditions, and the relevance of a robust gut microbiome to general health has generated significant interest in evaluating FMT therapy for a variety of systemic, non-gastrointestinal disorders. We now need to stop, reflect, and develop ways to harness the power of a therapy that might be capable of achieving in non-CDI conditions the success it enjoys in CDI. Orally available microbiota-based products, several of which are being developed currently, will facilitate treatment for *Clostridium difficile* infection and further expand the horizons of exciting therapeutic opportunities for numerous other non-CDI indications.
